# Modified Smith predictor-based fuzzy self-tuning FOPID controller for mean arterial pressure regulation

**DOI:** 10.1038/s41598-026-41657-2

**Published:** 2026-03-18

**Authors:** Hamed Khodadadi, Hamid Ghadiri, Ali Dehghani

**Affiliations:** 1 Department of Electrical Engineering, Kho.C., Islamic Azad University, Khomeinishahr, Iran; 2 Department of Electrical Engineering, Qa.C., Islamic Azad University, Qazvin, Iran

**Keywords:** Mean arterial pressure, MIMO time-delay system, Modified Smith predictor, Fuzzy logic self-tuning fractional-order PID controller, Computational biology and bioinformatics, Engineering

## Abstract

The simultaneous regulation of hemodynamic variables, particularly mean arterial pressure (MAP) and cardiac output (CO), is challenging due to time-varying delays, cross-coupling effects, and parameter uncertainty inherent in drug–response dynamics. A robust control framework is developed that integrates a fractional-order PID (FOPID) controller with a fuzzy logic self-tuning mechanism (FLST) and a modified Smith predictor (MSP) to enable adaptive gain scheduling and explicit compensation of bounded delays. The patient is modeled as a two-by-two first-order time-delay MIMO system actuated by sodium nitroprusside (SNP) and dopamine (DPM). Besides, transport and recirculation delays are assumed variable and conservatively bound at 60 s. While the inclusion of fractional orders provides additional degrees of freedom, resulting in improved transient performance and enhanced steady-state accuracy, the MSP maintains stability and tracking performance in the presence of time-varying delays. Extensive simulation studies under nominal and uncertain conditions demonstrate faster rise and settling times, reduced overshoot, and lower integral error indices than those of conventional PID and fractional-order PID controllers. Robustness to inter-patient variability in the presence of disturbances and noise measurement, and clinically acceptable performance, indicating feasibility for real-time closed-loop drug infusion. Overall, the proposed FLST–FOPID–MSP architecture offers a promising and safety-oriented solution for automated MAP and CO regulation and motivates future comparisons with predictive and -based control strategies.

## Introduction

As a hydraulic system, the cardiovascular is known as one of the most important cycles of the human body’s life. In this system, blood flow is governed by principles of pressure and fluid dynamics^1^. Regulation of the average blood pressure throughout a cardiac cycle, named mean arterial pressure, is vital to preventing acute life-threatening conditions^2^. In other words, reducing fluctuations in MAP can significantly decrease the risk of surgical complications^3^. While the cardiac output is one of the parameters directly influences MAP^2^, both MAP and CO are critical variables utilized by clinicians to assess a patient’s hemodynamic status^1^. These hemodynamic variables can be maintained in a suitable operating range by intravenous infusing several drugs^3,4^. The importance of controlling the hemodynamic variables is highlighted for cardiac diseases patient during the surgery and postsurgical patient recovery period^5,6^.

Sodium nitroprusside and phenylephrine (PNP) are typically used to control MAP, whereas dopamine and intravenous fluids are administered to enhance CO^4^. An MAP above the upper threshold may increase the risk of metabolic syndrome, cerebral hyperperfusion, cerebral edema, and exacerbation of pre-existing neurological damage. Conversely, an MAP below the permissible lower limit can lead to ischemia and worsen brain injury^7^. In cases of cardiac arrest, hypoxic-ischemic brain damage is a major cause of mortality^2,8^. Thus, maintaining MAP and CO within optimal bounds is essential for minimizing surgical risks and improving survival outcomes^2,7^.

On the other hand, accurate control of drug infusion rates is particularly challenging due to inter-patient variability in drug sensitivity. For instance, excessive SNP administration may result in toxic side effects. Therefore, determining the appropriate drug dosage requires precise control, which is difficult to achieve manually^2,9^. Automatic drug delivery in the form of a feedback control system offers a viable solution for real-time fine-tuning the drug infusion rate based on the measurements of MAP and CO. This can enhance the quality of surgical care and reduce the risks associated with manual intervention^2,3,9^. Consequently, the regulation of blood pressure and related hemodynamic parameters has attracted significant research interest.

The surveyed studies show that Various automatic control strategies have been developed for this purpose. These studies differ in the number of controlled variables (e.g., MAP alone vs. both MAP and CO) and in the number and type of drugs administered^3^. The traditional PID controller is the primary selection to control the hemodynamic variables^10,11^, albeit it has a weekly performance to control nonlinear, uncertain, and coupled systems. Therefore, to address these limitations, researchers have suggested various intelligent techniques such as fuzzy logic^12,13^, deep learning neural network^14^, and adaptive active disturbance rejection control^15^. Although these methods have shown improved performance over conventional PID, PID controllers are still of interest due to their simplicity and effectiveness. Furthermore, the other techniques such as optimized PID using genetic algorithm (GA)^16^, model predictive control (MPC)^2,17,18^, internal model control (IMC)^19^, optimal IMC^10,20^, robust control^1,18^, model reference adaptive control (MRAC)^3,21^, flatness-based control^5^, fractional order control^22^, sliding mode control^21^, and self-tuning control^23^ have also been proposed for hemodynamic regulation.

Among these, researches demonstrate that fractional-order controllers have demonstrated superior performance than integer-order controllers^6,24,25^. In the fractional-order PID controller, two new parameters consist of $\:\lambda\:$, and $\:\mu\:$, as the integral and derivative fractional-order, are inserted into the controller^26^. On the other hand, the delay phenomenon that occurs in many physical systems causes poor performance and instability in the system^27,28^. Traditional PID controllers are particularly vulnerable to such delays and require return if system parameters vary. In recent years, substantial progress has been reported in the development of advanced adaptive and intelligent control strategies for biomedical systems subject to time delays and patient-specific uncertainties. Adaptive model predictive control schemes have been proposed to explicitly address multivariable interactions and operational constraints in hemodynamic regulation problems^29,30^. In addition, learning-based adaptive controllers, including neural-network-assisted and reinforcement-learning-based approaches, have demonstrated an enhanced ability to capture nonlinear pharmacodynamic relationships and compensate for uncertain and time-varying delays^31,32^. Parallel advances in fractional-order adaptive control and delay-dependent stability analysis have further shown improved robustness and flexibility over conventional integer-order methods when applied to physiological systems^33–35^. Collectively, these developments emphasize the importance of integrating adaptive mechanisms with robust delay compensation to achieve reliable closed-loop performance in complex multivariable drug infusion systems.

In practical control applications, time delays are seldom constant and often exhibit time-varying behavior governed by stochastic or bounded characteristics. Recent studies have demonstrated that explicitly incorporating delay information into control design can significantly improve system stability, robustness, and performance, particularly in the presence of uncertainty and network-induced delays. Delay-distribution-dependent stability and control conditions have been formulated using Lyapunov-based methods and convex optimization techniques, which yield fewer conservative results than delay-independent approaches^36,37^. Such frameworks have been successfully applied to complex large-scale systems, including fuzzy systems and power networks, where delays follow known probabilistic or bounded profiles. Motivated by these findings, time-varying delays are treated in this work as an inherent characteristic of drug infusion dynamics in postoperative patients. Although a full probabilistic delay-distribution modeling framework is beyond the scope of the present study, the proposed modified Smith predictor is designed to robustly compensate for uncertain and time-varying delays. In this case, reliable performance under realistic physiological delay variations will be guaranteed. To address these issues, intelligent control methods like adaptive fuzzy^38^, fuzzy model reference adaptive^39,40^, and self-tuning fuzzy control^41,42^ have been explored.

This paper presents a new approach for automatic closed-loop SNP and DPM administration to regulate both MAP and CO within optimal range. The assumed model for describing the relation between the infusion rate of drugs and hemodynamic variables is selected from^3^ as a two-by-two first-order time-delayed transfer function. The existence of time delay, MIMO structure, modeling uncertainty, time-varying parameters, noise and output disturbances are the main challenges in this system. To address these, a self-tuning fractional-order PID controller based on the fuzzy logic part is proposed. The robust structure of the fuzzy logic controller is incorporated into the adaptive specification of the self-tuning controller to handle the wide range sensitivity and reaction times to drugs for different patients. Besides, employing the fractional calculus provides two extra degrees of freedom (DOF), and flexibility leads to better performance in transient characteristic and steady-state responses. Additionally, to mitigate the adverse effects of uncertain and time-varying delays, a modified Smith predictor (SP) is integrated into the control structure. Hence, this results in a fuzzy logic self-tuning fractional-order PID controller enhanced with an MSP and makes the proposed approach as the FLST-FOPID based on the MSP. In addition to MRAC, the proposed controller is benchmarked against several widely adopted advanced control strategies, including delay-aware MPC, ADRC, and robust H∞ control, to provide a comprehensive and fair performance evaluation.

The main scientific contributions of this work can be summarized in two key advancements. First, a unified adaptive control framework is developed for the simultaneous regulation of mean arterial pressure and cardiac output in postsurgical cardiac patients. The proposed framework integrates a fuzzy logic self-tuning fractional-order PID controller with a modified Smith predictor. The fractional-order structure introduces additional degrees of freedom for shaping both transient and steady-state responses, while the fuzzy self-tuning mechanism enables real-time adaptation of controller gains in the presence of patient-specific uncertainties and nonlinear drug dynamics. Moreover, the MSP is employed to effectively compensate for uncertain and time-varying drug-response delays, thereby preserving closed-loop stability and performance in a multivariable time-delay setting. Second, extensive simulation studies conducted under both nominal and uncertain patient conditions demonstrate the superior performance of the proposed FLST–FOPID–MSP approach compared with existing robust and intelligent control strategies. Improvements are observed in tracking accuracy, disturbance rejection capability, noise attenuation, and robustness to inter-patient variability, highlighting the potential of the proposed method for reliable real-time implementation in automated drug infusion systems.

This paper is organized as follows: Sect. 2 outlines the patient model in both cases of considering the MAP regulation alone and in combination with CO. Section 3 introduces the control strategy, its components, and the rule of their combination. The principle and design steps of FOPID controller, fuzzy logic, smith predictor, modified Smith predictor, and FLST–FOPID controller based on MSP will be discussed in this section. In Sect. 4, the simulation results of the proposed controller for the nominal and uncertain conditions of the patient model are illustrated and compared with the other controllers. Finally, the concluding remarks are given in Sect. 5.

## Mathematical model of the patient for mean arterial pressure regulation

The relationship between the MAP and the SNP infusion rate can be described as follows^39^:

1 $$\:{P}_{\varDelta\:}\left(s\right)={k}_{p}\frac{{e}^{{-T}_{{i}^{s}}}\left(1+\alpha\:{e}^{{-L}_{{i}^{s}}}\right)}{1+\tau\:s}L\left(s\right)\:$$

where $\:{P}_{\varDelta\:}\left(s\right)$ denotes the MAP change, *L(s)* stands the infusion rate, $\:{k}_{p}$ and $\:\alpha\:$ are the patient’s sensitivity to the drug and the recirculation constant reflecting the effect of the recirculated drug on the patient’s MAP, respectively. The ranges of these parameters are $\:0.25\le\:{k}_{p}\le\:8$ and $\:0\le\:\alpha\:\le\:1$. Also, $\:{T}_{i}$ and $\:{L}_{i}$ indicate the initial transport and recirculation dead time and are in the range of $\:20\le\:{T}_{i}\:,{L}_{i}\le\:60\:sec$. Besides, $\:\tau\:$ is devoted to the system time constant ($\:20\le\:\tau\:\le\:60\:sec$)^39^.

Despite its utility, the model described in Eq. 1 has two significant challenges. Both MAP and CO are the essential variables of clinicians’ cardiovascular system for describing the system’s operation^1^. Considering only one of these variables (as stated in Eq. 1) cannot present a complete description of the system. Second, Eq. 1 has two delays. The presence of two distinct delay components increases the complexity of control system design, may lead to degrading controller performance.

Therefore, to overcome these drawbacks, a two-input, two-output model is employed in which the MAP and CO are the system outputs, and the SNP and DPM composed the system inputs. The relation between these inputs and outputs is described by a linear first-order transfer function with dead time, presented in Eqs. 2, 3. Increasing the CO and decreasing the MAP of the patient to the desired values are the primary purposes of controlling this system. The effects of the input variations on the system outputs are as follows.

The interaction effects among the variables are clinically significant. An increase in SNP dosage typically reduces MAP while simultaneously increasing CO. Conversely, DPM administration tends to elevate both MAP and CO. The drug infusion rates are expressed in mg/min·kg, CO in mL/min·kg, and MAP in mmHg.

2 $$\:CO={G}_{11}\times\:DPM+{G}_{12}\times\:SNP+{D}_{1}$$,

3 $$\:MAP\:={G}_{21}\times\:DPM+{G}_{22}\times\:SNP+{D}_{2}.$$

where

4 $$\:{G}_{ij}=\frac{{{K}_{ij}\:e}^{{-T}_{{ij}^{s}}}}{{\tau\:}_{{ij}^{s}}+1}.\:\:\:\:\:\:\:\:\:\:\:\:\:\:\:\:\:i.j=1.2.$$

In Eq. 4, $\:{K}_{ij}\:$ indicates the model gain, $\:{\tau\:}_{ij}$ is devoted to the time constant and $\:{\mathrm{T}}_{ij}$ indicates time delay. Moreover, the effect of disturbances on the system outputs is shown by the $\:{D}_{1}\:$and $\:{D}_{2}$^3^. The numerical values and ranges of the system parameters are summarized in Table 1. These parameters are derived from empirical data in^3^, and reflect both nominal and patient-dependent variability. Furthermore, pharmacological safety constraints restrict the SNP and DPM infusion rates to within the ranges $\:0\le\:\mathrm{D}\mathrm{P}\mathrm{M}\le\:6$ and $\:0\le\:\mathrm{S}\mathrm{N}\mathrm{P}\le\:10$.

## Controller design

### Fractional-order PID controller

Due to the simple structure of the PID controllers, ease of implementation, and effective performance in a broad range of applications, they are used in almost all systems. However, conventional PID controllers are inherently limited to integer-order calculus that led to potentially inadequately capture the complex dynamics of physiological systems. To address these limitations, fractional-order PID controllers extend the classical PID framework via employing fractional calculus. This generalization allows the integral and derivative orders to assume non-integer values. By this approach, more accurate modeling and improved control performance are provided for systems exhibiting anomalous or distributed-order dynamics.

**Table 1:** Nominal values and permissible ranges of system parameters^3^.

Parameters	Value	Ranges
$\:{\mathrm{K}}_{11}$	5	1 to 12
$\:{{\uptau\:}}_{11}$	300	70 to 600
$\:{\mathrm{T}}_{11}$	60	15 to 60
$\:{\mathrm{K}}_{12}$	12	− 15 to 25
$\:{{\uptau\:}}_{12}$	150	70 to 600
$\:{\mathrm{T}}_{12}$	50	15 to 60
$\:{\mathrm{K}}_{21}$	3	0 to 9
$\:{{\uptau\:}}_{21}$	40	30 to 60
$\:{\mathrm{T}}_{21}$	60	15 to 60
$\:{\mathrm{K}}_{22}$	− 15	− 1 to − 50
$\:{{\uptau\:}}_{22}$	40	30 to 60
$\:{\mathrm{T}}_{22}$	50	15 to 60

**Fig. 1:**
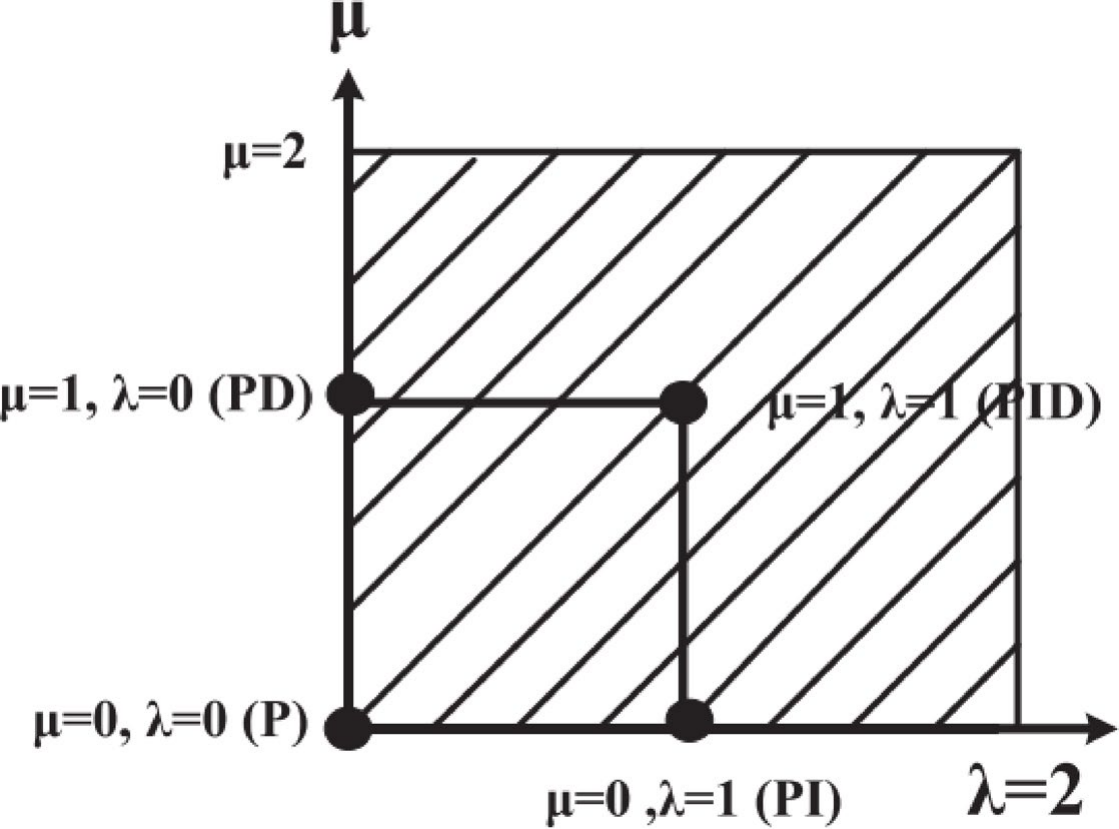
Fractional PID controller converge^26^.

**Fig. 2:**
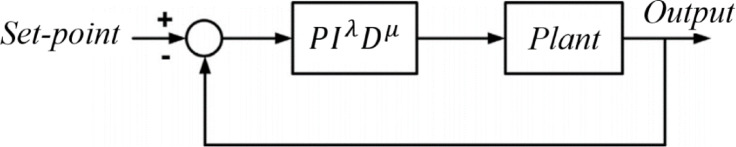
Block diagram of a Fractional PID^26^.

The FOPID controller introduces two additional tuning parameters: the fractional integral order $\:\lambda\:$ and the fractional derivative order $\:\mu\:$, alongside the conventional gains of $\:{K}_{p}$, $\:{K}_{i}$, and $\:{K}_{d}$. This increased flexibility contributions the controller two extra degrees of freedom, help the controller for enhanced shaping of transient and steady-state responses. In other words, the conventional PID controller can be assumed as a special case of a fractional controller when $\:\lambda\:$ and $\:\mu\:$ are equal to one. The fractional controller convergence and its structure in a closed-loop system are shown in Figs. 1 and 2, respectively^26^.

The FOPID transfer function is written as follows^26^.

5 $$\:C\left(s\right)={K}_{p}+{K}_{i}{s}^{-\lambda\:}+{K}_{d}{s}^{\mu\:}$$

This compensator computes the control signal based on (6).

6 $$\:u\left(t\right)=\left({K}_{p}+{K}_{i}{D}^{-\lambda\:}+{K}_{d}{D}^{\mu\:}\right)e\left(t\right)$$

Where$\:\:u\left(t\right)$, $\:e\left(t\right)=r\left(t\right)-y\left(t\right)$, $\:r\left(t\right)$ and $\:y\left(t\right)$ indicate the command, error, reference, and output signals, respectively. The controller parameters consist of proportional, integral, and derivative gains are demonstrated as $\:{K}_{p}.\:{K}_{i}$ and $\:{K}_{d}$^26^. Via tuning $\:\lambda\:$ and $\:\mu\:$, the controller can better accommodate the non-integer dynamics often observed in biomedical systems and therefore, providing the improved robustness, and disturbance rejection^38^.

###  Fuzzy logic

Fuzzy logic is one of the artificial intelligence methods that can systematically formulate human knowledge and linguistic imprecision through mathematical constructs^43^. Fuzzy Sets are useful in establishing imprecise conditions and are very suitable for medical decision-making especially when system behavior lacks precise mathematical definitions. Fuzzy Inference System (FIS), as the core architecture of fuzzy logic-based control systems, comprises several main parts consisting of fuzzifier, rule base, inference engine, and defuzzifier^44^. While during the fuzzification, the crisp input values maps into fuzzy sets, defuzzification performs the opposite task. By combining the fuzzy outputs obtained from the processing stage, one crisp value is calculated. The processing stage is the derivation of the FIS based on the fuzzy rule base.

This rule-based structure enables fuzzy controllers to handle imprecise data and uncertain environments. This feature makes them highly applicable in medical decision-making, especially where patient responses vary significantly and cannot be modeled accurately by traditional mathematical techniques.

### Fuzzy logic self-tuning

To enhance the adaptability of the fuzzy logic controller in dynamic or uncertain environments, a self-tuning mechanism is incorporated into the system. This approach enables the controller to respond effectively to parameter variations, disturbances, and model inaccuracies without the need for external intervention. Employing the Mamdani’s minimum for the fuzzy implication and the center average for the defuzzification scheme, the fuzzy control law is presented as (7):

7 $$\:{u}_{1}=\frac{\sum\:_{i.j}[\left({\mu\:}_{{E}_{i}}\left({e}_{1}\right)\cap\:{\mu\:}_{{E}_{j}}\left({e}_{2}\right)\right).{U}_{n(i.j)}]}{\sum\:_{i.j}\left({\mu\:}_{{E}_{i}}\left({e}_{1}\right)\cap\:{\mu\:}_{{E}_{j}}\left({e}_{2}\right)\right)}$$

where $\:{\mu\:}_{{E}_{i}}\left({e}_{1}\right)\cap\:{\mu\:}_{{E}_{j}}\left({e}_{2}\right)\:$ indicates the intersection of two fuzzy sets, defined as the “min” operation (Eq. 8)

8 $$\:{\mu\:}_{{E}_{i}}\left({e}_{1}\right)\cap\:{\mu\:}_{{E}_{j}}\left({e}_{2}\right)=\mathrm{m}\mathrm{i}\mathrm{n}[{\mu\:}_{{E}_{i}}\left({e}_{1}\right).{\mu\:}_{{E}_{j}}\left({e}_{2}\right)]\:$$

Fuzzy if-then rule who builds the fuzzy control rule in a fuzzy logic controller can be represented as (9).

9 $$\:R:if\:{e}_{1}\:is\:{E}_{i}\:and\:{e}_{2}\:is\:{E}_{j}\:Then\:{u}_{1}\:is\:{U}_{n(i.j)}$$

In (9), $\:{e}_{1}$ and $\:{e}_{2}$ are the error $\:e\left(k\right)$ and its change $\:\varDelta\:e\left(k\right)$. Also, $\:{E}_{i}$, $\:{E}_{j}$ and $\:{U}_{n(i.j)}\:$denote the linguistic variables of $\:{e}_{1}$, $\:{e}_{2}$ and $\:{u}_{1}$, respectively. Besides, $\:n(i.j)$ is assumed as a function whose value at $\:i$ and$\:\:j$ is an integer^34,35^. A self-tuning structure is embedded into the fuzzy controller for compensating the disturbance effects, mismatch dynamics, and uncertainty on system parameters leads Eq. 9 converted to (10).

10 $$\:R:if\:{e}_{1}\:is\:{E}_{i}\:and\:{e}_{2}\:is\:{E}_{j}\:Then\:C\:is\:{C}_{m(i.j)}$$

where $\:C$ and $\:{C}_{m(i.j)}$ are the changes in the center of the output Membership Function (MF) and its linguistic variable. Similar to $\:n(i.j)$, assume that $\:m(i.j)$ represents any function with an integer value^42,45^. Applying the Mamdani’s minimum and the center average for fuzzy implication and defuzzification, the center of MF output for the self-tuning fuzzy controller is tuned as:

11 $$\:{U}_{n(i.j)}={U}_{n(i.j)}+\frac{\sum\:_{i.j}[\left({\mu\:}_{{E}_{i}}\left({e}_{1}\right)\cap\:{\mu\:}_{{E}_{j}}\left({e}_{2}\right)\right).{C}_{m(i.j)}]}{\sum\:_{i.j}\left({\mu\:}_{{E}_{i}}\left({e}_{1}\right)\cap\:{\mu\:}_{{E}_{j}}\left({e}_{2}\right)\right)}$$

This adaptive update allows the controller to refine its behavior by shifting the center of output membership functions in response to changing system dynamics. In this way, the fuzzy controller can maintain high performance even in the presence of parameter uncertainty or external disturbances. Moreover, this tuning strategy can be extended to adjust scaling factors or membership shapes, enhancing the robustness and flexibility of the control system in real-time applications^46,47^.

### Smith predictor

Time delays arise in many industrial processes as a consequence of various phenomena. Time delays affect systems’ performance because they can lead to poor system response, prevent high controller gain, and even caused instability. The presence of delay in the control loops has two chief consequences: the analysis and designing of the controllers make very complicated, and the desired performance hardly reached. Deadtime compensators can be utilized to improve the classical controllers’ performance for processes with delay^48,49^.

**Fig. 3:**
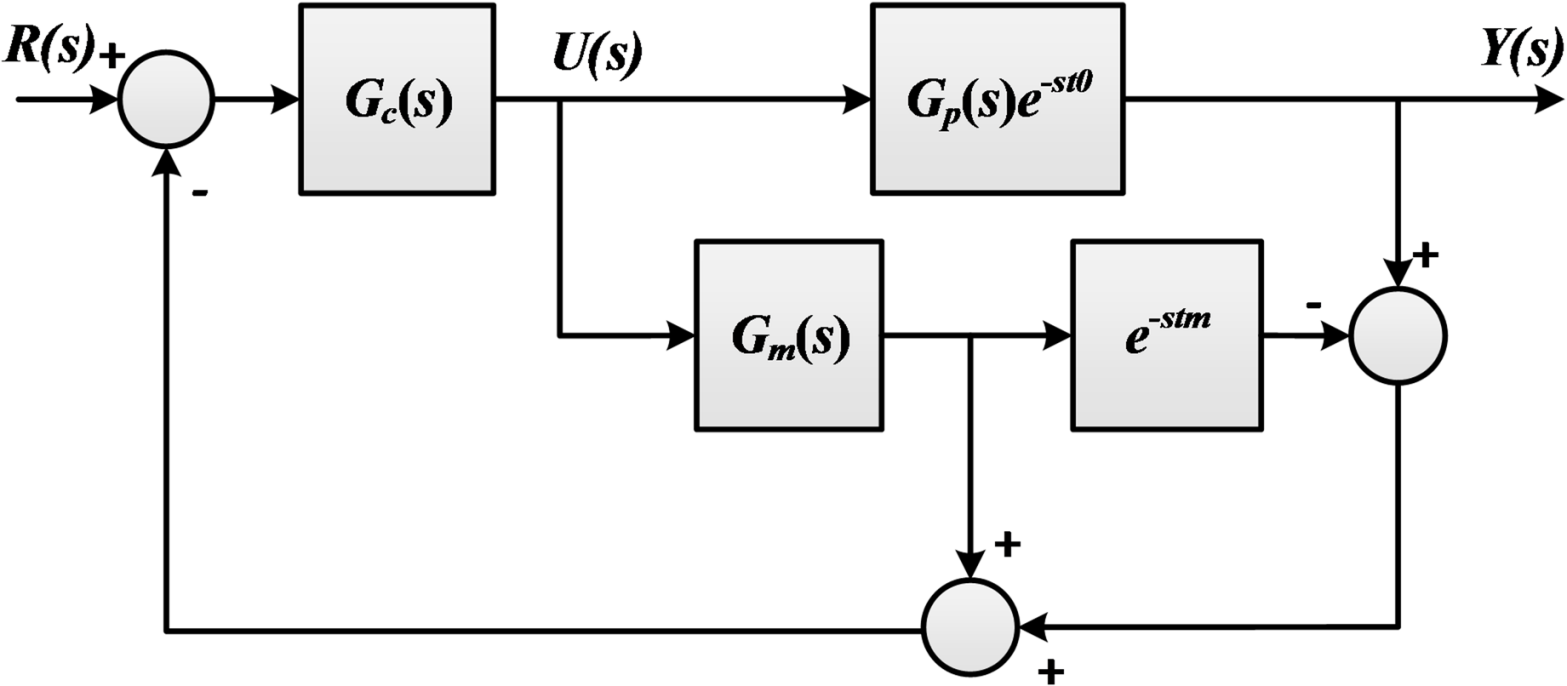
Conventional Smith predictor structure^51^.

The SP can be considered as one of the compensation methods, especially applied in the industry. The core advantage of the SP lies in its ability to effectively eliminate the delay component from the system’s characteristic equation, thereby simplifying the controller design and improving performance^49^. However, due to the structural complexity of most industrial processes, especially in MIMO cases, control becomes more challenging when delays are present. Dead time not only reduces the system phase margin but also makes analysis and control design more intricate and less robust^50^. The structure of the conventional Smith Predictor is illustrated in Fig. 3^51^. In this figure, $\:{G}_{C}\left(s\right)$ is controller, $\:{G}_{P}\left(s\right)$ represent the plant transfer function without delay, $\:{G}_{m}\left(s\right)$ denotes the model transfer function without delay, and $\:{e}^{-s{t}_{0}}$ and $\:{e}^{-s{t}_{m}}$ are the system time delay and its estimation. Besides, $\:y$ and $\:r$ indicate the system output and reference input, respectively.

### Modified Smith predictor

**Fig. 4:**
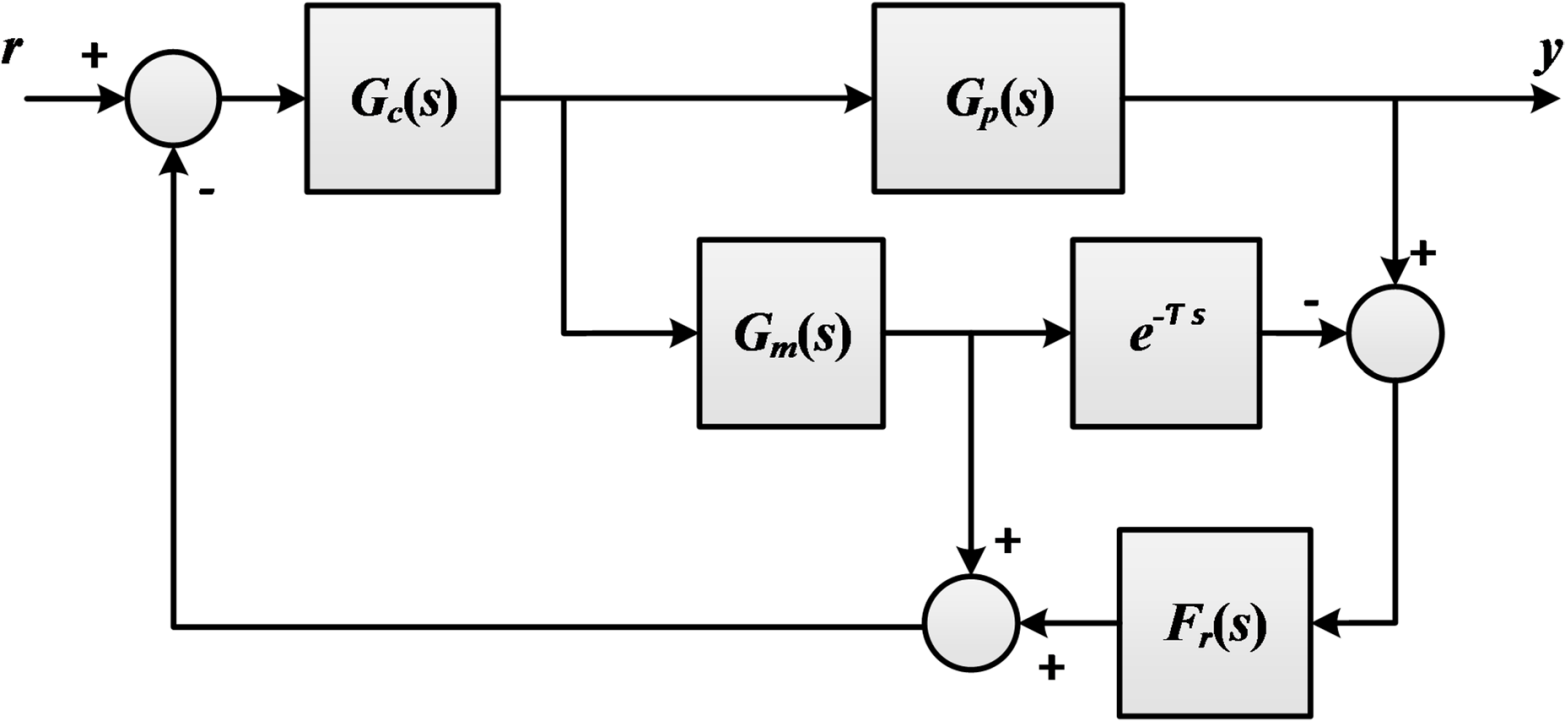
Modified Smith predictor structure^51^.

Although the standard form of the SP can effectively compensate for time delay, it relies on an accurate mathematical model of the system. However, due to the substantial delays and time-varying parameters observed across different patients, obtaining an exact model of the physiological system is highly challenging. A modified version of the Smith predictor introduced in^51^ is utilized here to address this issue. In this approach, by adding the first-order filter to the Smith structure’s standard form, the system’s robustness and adaptability are improved. As can be seen in Fig. 4, the inserted filter is described as $\:{F}_{r}\left(s\right)=\frac{1}{{T}_{f}s+1}$ in which, $\:{T}_{f}.$ $\:{T}_{m}.\:{K}_{c}\:\mathrm{a}\mathrm{n}\mathrm{d}\:{K}_{m}$ are the filter time constant, predictor constant, $\:{G}_{c}\left(s\right)$ gain and SP gain, respectively. The closed-loop transfer function of Fig. 4 is obtained as:

12 $$\:\frac{Y\left(s\right)}{R\left(s\right)}=\frac{{G}_{c}\left(s\right){G}_{p}\left(s\right){e}^{-\tau\:s}}{1+{G}_{c}\left(s\right){G}_{m}\left(s\right)+{G}_{c}\left(s\right){(G}_{p}\left(s\right){e}^{-\tau\:s}-{G}_{m}\left(s\right){e}^{-{\tau\:}_{m}s})\frac{1}{{T}_{f}s+1}}$$

Comparison between Eq. 12 and the standard form of SP demonstrate that if $\:{G}_{p}\left(s\right)={G}_{m}\left(s\right).\:\tau\:={\tau\:}_{m}$, the MSP is completely acting like SP by a pure lag time shift in the output response. If the system has some uncertainties in the model and time delay means $\:{G}_{p}\left(s\right)\ne\:{G}_{m}\left(s\right).\:\tau\:\ne\:{\tau\:}_{m}$, the filter link makes the main feedback channel and acts as a buffer to the mismatch predictor model. The filter time constant can be tuned to improve the performance and stability of the control system. For $\:{T}_{f}=0$, the MSP is converted to the SP.

### Theoretical analysis of MSP filter time constant and robustness

Within the proposed FLST–FOPID framework, the MSP incorporates a first-order low-pass filter of the form

13 $$\:F\left(s\right)=\frac{1}{{T}_{f}s+1}$$

where the filter time constant $\:{T}_{f}$ plays a critical role in determining closed-loop stability and robustness in the presence of time-delay uncertainty.

#### Stability boundaries for $\:{\boldsymbol{T}}_{\boldsymbol{f}}$

By applying the small-gain theorem to the uncertain MIMO time-delay system, closed-loop stability is guaranteed if the following condition is satisfied:

14 $$\:sup\left|{G}_{c}\left(j\omega\:\right){G}_{p}\left(j\omega\:\right)\frac{{e}^{-j\omega\:\tau\:}-F\left(j\omega\:\right)}{1+{G}_{c}\left(j\omega\:\right){G}_{p}\left(j\omega\:\right)F\left(j\omega\:\right)}\right|<1$$

where $\:{G}_{p}\left(s\right)$ is the patient model process, $\:{G}_{c}\left(s\right)$ is the FLST-FOPID controller, and $\:\tau\:\le\:60\:s$ is the bounded time delay. Numerical evaluation yields a practical range:

15 $$\:8\:sec<{T}_{f}<20\:sec$$

Values below 8 s tend to amplify high-frequency noise, while values above 20 s introduce excessive phase lag.

#### Robustness assessment

Robustness properties are further examined through sensitivity function analysis, defined as

16 $$\:S\left(j\omega\:\right)=\frac{1}{1+{G}_{c}\left(j\omega\:\right){G}_{p}\left(j\omega\:\right)F\left(j\omega\:\right)}$$

across the $\:{T}_{f}$ range indicates optimal robustness at $\:{T}_{f}\approx\:\:12-15\:sec$, where sensitivity peaks are minimized without degrading transient performance.

#### Practical guidelines

Based on the theoretical analysis and numerical evaluations, practical guidelines for selecting the MSP filter time constant are summarized in Table 2.

**Table 2:** Practical guidelines for selecting the MSP filter time constant.

Parameter	Recommended range	Purpose
$\:{T}_{f}$	8–20 s	Balance robustness and response speed
Optimal $\:{T}_{f}$	12–15 s	Maximizes robustness margin

### Fuzzy logic self-tuning fractional-order PID controller based on modified smith predictor

**Fig. 5:**
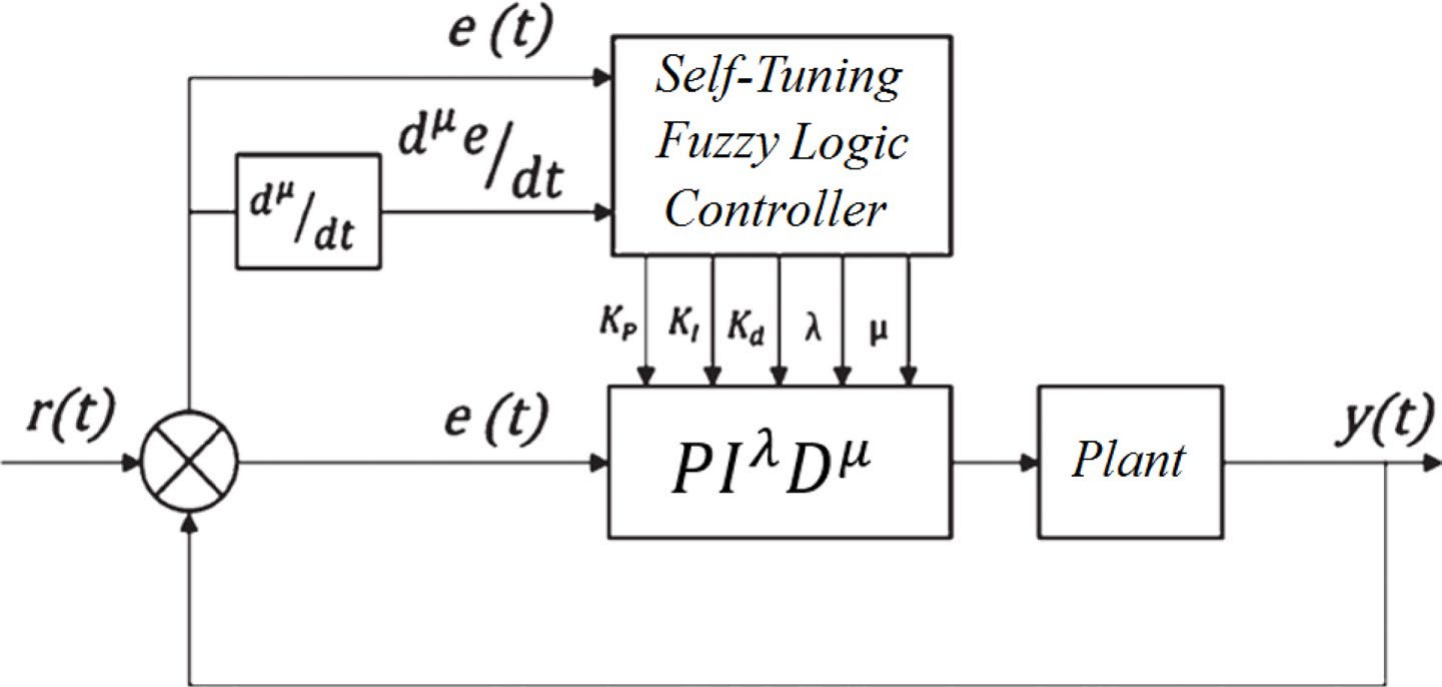
Structure of the FLST-FOPID controller.

**Fig. 6:**
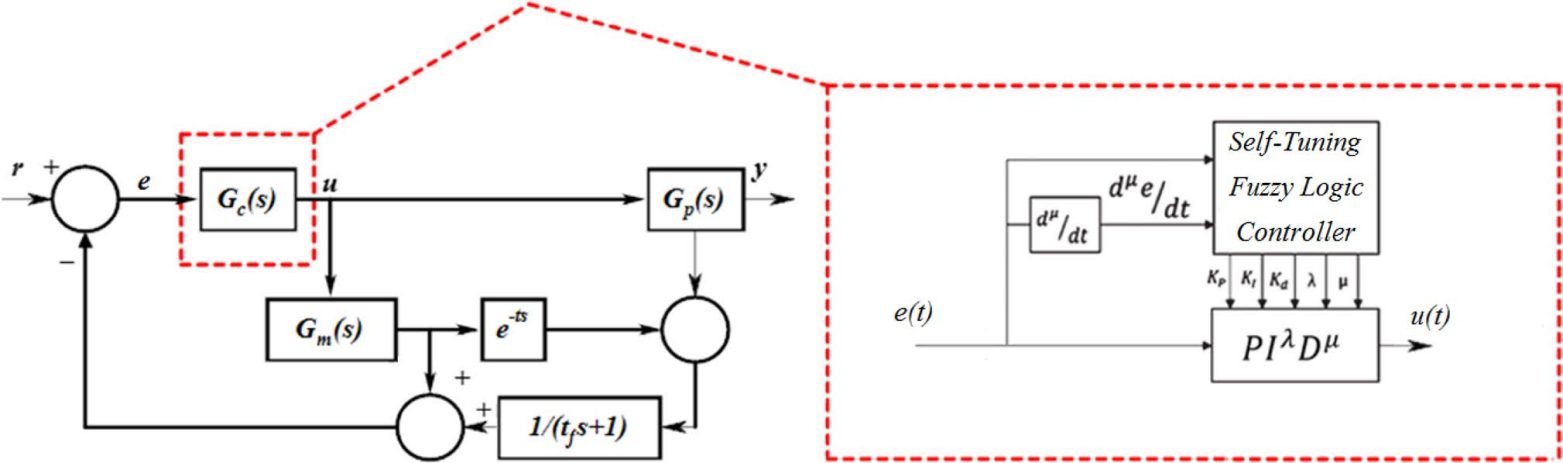
Structure of the proposed FLST-FOPID controller based on the MSP.

The method proposed in this study for the simultaneous regulation of both MAP and CO integrates a self-tuning fuzzy logic mechanism with a FOPID controller. In contrast to the classical FOPID controller, the parameters of the PID terms in this approach are not fixed; instead, they are dynamically adjusted using a FLST strategy. The overall structure of the FLST-FOPID controller is depicted in Fig. 5. Furthermore, to compensate for the system’s inherent time delay, the FLST-FOPID controller is integrated with the MSP. The complete structure of the proposed control scheme which is referred to as the FLST-FOPID controller based on the MSP, is illustrated in Fig. 6.

### Allowable upper bound of time-varying delays

In the considered hemodynamic regulation system, patient dynamics are modeled as a first-order multivariable process. According to the patient model adopted from^3^, the system delay varies within a bounded interval, as summarized in Table 1. Let $\:T\left(t\right)$ denote the actual time-varying delay of the system, and $\:\widehat{T}$ represent the nominal delay used in the MSP. The delay mismatch can therefore be defined as:

17 $$\:\varDelta\:T\left(t\right)\:=\:T\left(t\right)-\:\widehat{T}$$

To ensure closed-loop stability, the time-varying delay is assumed to satisfy the following admissibility conditions:

18 $$\:0\:\le\:T\left(t\right)\le\:{T}_{max},\hspace{1em}\left|dT\right(t)/dt|\le\:\mu\:<1$$

where $\:{T}_{max}$ is the allowable upper bound of the delay, and $\:\mu\:$ denotes the maximum rate of delay variation.

Within the proposed control structure, the MSP compensates for the nominal delay, while the embedded first-order filter provides a robustness margin by attenuating the adverse effects of delay mismatch and modeling uncertainty. Based on classical Smith predictor stability principles, the admissible delay bound is directly related to the bandwidth of the equivalent delay-free closed-loop system. Preservation of an adequate phase margin and stable operation requires that the maximum allowable delay satisfy

19 $$\:{T}_{max}<\frac{1}{{\omega\:}_{c}}$$

where $\:{\omega\:}_{c}$ is the closed-loop crossover frequency.

In this study, by applying the system parameters listed in Table 1, where physiological delays are reported to range from 15 s to 60 s, and considering the selected MSP filter time constant, the maximum admissible delay is conservatively chosen as $\:{T}_{\mathrm{m}\mathrm{a}\mathrm{x}}=60\mathrm{\:s}$. This bound fully encompasses clinically reported delay variations of the adopted patient model and ensures stable closed-loop performance of the proposed FLST–FOPID controller under bounded and slowly time-varying delays.

## Simulation results and discussion

###  Nominal system

In this section, the FLST-FOPID based on MSP controller is designed for the simultaneous regulation of MAP and CO in the postsurgical patients and its simulation results will be presented. Employing the introduced model of the system (2–4) and the numerical values for the system parameters (Table 1), make the final model which is used in the simulation (Eq. 13).

20 $$\:\left[\begin{array}{c}\:CO\\\:MAP\end{array}\right]=\left[\begin{array}{cc}\frac{5{\mathrm{e}}^{-60\mathrm{s}}}{\:300\mathrm{s}+1}\:&\:\frac{12{\mathrm{e}}^{-50\mathrm{s}}}{150\mathrm{s}+1}\:\\\:\frac{{3\mathrm{e}}^{-60\mathrm{s}}\:}{40\mathrm{s}+1}\:&\:\frac{-15{\mathrm{e}}^{-50\mathrm{s}}}{40\mathrm{s}+1}\:\end{array}\right]\left[\begin{array}{c}\:DPM\\\:SNP\end{array}\right]$$

To implement the fuzzy FOPID controller for the outputs (MAP and CO); the output error and its derivate are considered as the inputs of the fuzzy system and the FOPID parameters (i.e. $\:{K}_{p}.\:{K}_{i}$ and $\:{K}_{d}$) obtained from the fuzzy inference are assumed as the outputs.

**Fig. 7:**
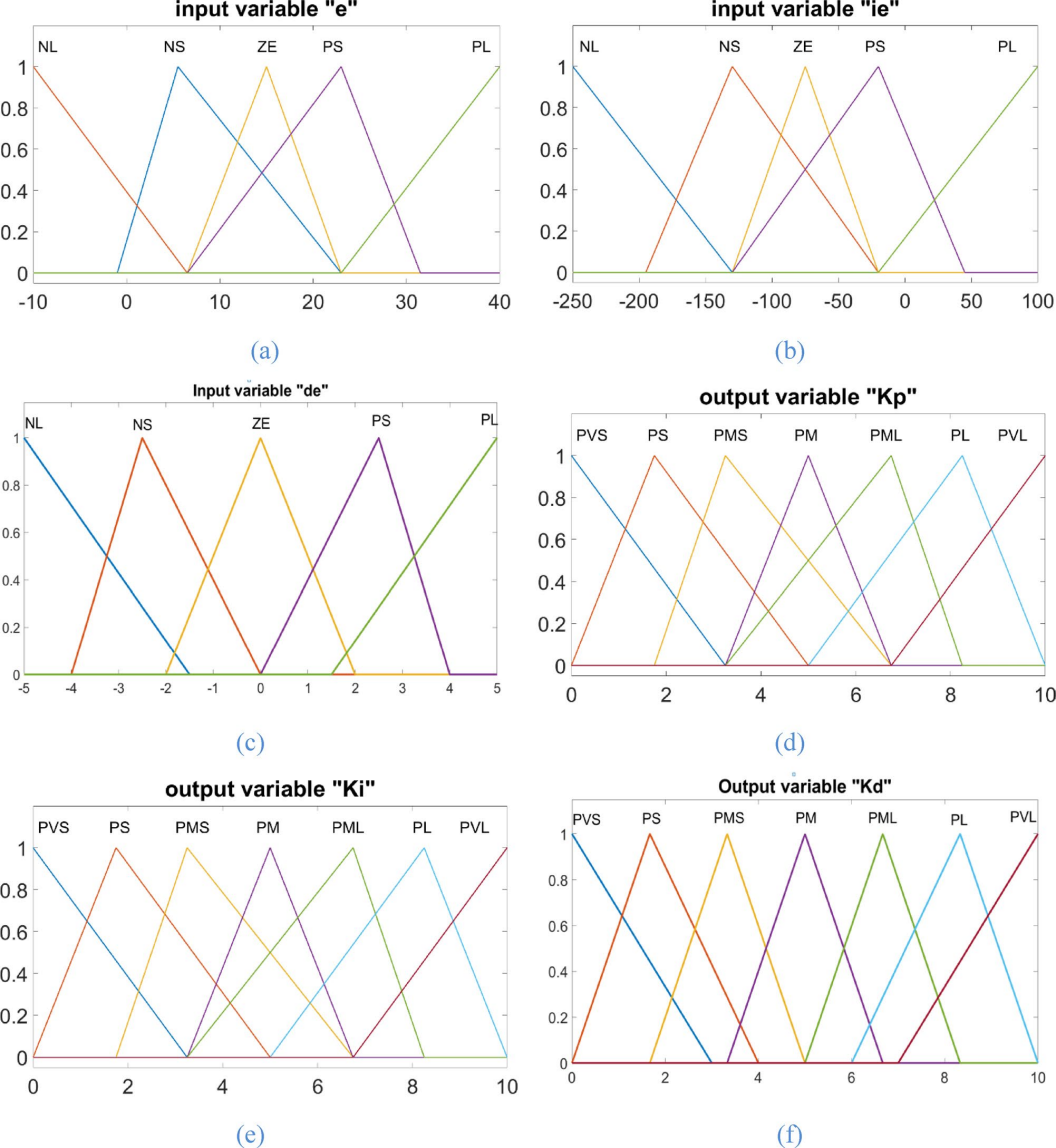
Membership functions of the fuzzy inputs and outputs for the MAP regulation.

Triangular membership functions are employed for the fuzzy sets. The input variables (error and change of error) use five linguistic levels: zero (ZE), negative large (NL), negative small (NS), positive small (PS), and positive large (PL). The output variables utilize seven levels: positive very small (PVS), positive small (PS), positive medium small (PMS), positive medium (PM), positive medium large (PML), positive large (PL), and positive very large (PVL). Figure 7 illustrates the membership functions used for the fuzzy inputs and two of the outputs, $\:{K}_{p}$ and $\:{K}_{i}\:$in MAP regulation.

The fuzzy rule base is summarized in Tables 3, 4 and 5, representing the inference matrices for computing $\:{K}_{p}$, $\:{K}_{i}$ and $\:{K}_{d}$.

**Table 3:** Fuzzy rules for $\:{K}_{p}$ computation.

CE	E				
	NL	NS	ZE	PS	PL
NL	PVL	PVL	PVL	PVL	PVL
NS	PML	PML	PML	PML	PML
ZE	PVS	PVS	PS	PMS	PMS
PS	PML	PML	PML	PM	PM
PL	PVL	PVL	PVL	PVL	PVL

**Table 4:** Fuzzy rules for $\:{K}_{i}$ computation.

CE	E				
	NL	NS	ZE	PS	PL
NL	PM	PM	PM	PM	PM
NS	PMS	PMS	PMS	PMS	PMS
ZE	PS	PS	PS	PS	PS
PS	PMS	PMS	PMS	PMS	PMS
PL	PM	PM	PM	PM	PM

**Table 5:** Fuzzy rules for $\:{K}_{d}$ computation.

CE	E				
	NL	NS	ZE	PS	PL
NL	PVL	PVL	PVL	PVL	PVL
NS	PMS	PMS	PMS	PMS	PMS
ZE	PS	PS	PVS	PS	PS
PS	PMS	PMS	PMS	PMS	PMS
PL	PM	PM	PM	PM	PM

Following the rule-based computation, the center of each output membership function is tuned in accordance with Eq. (11). The finalized controller structure is implemented as shown in Fig. 6 for regulating the nominal system. For the simulation, the reference values of MAP and CO are set at 120 mmHg and 100 ml/min·kg, respectively. The initial conditions are assumed to be 140 mmHg for MAP and 80 ml/min·kg for CO. Thus, the control objective is to reduce MAP by 20 mmHg and simultaneously increase CO by 20 ml/min·kg.

**Fig. 8:**
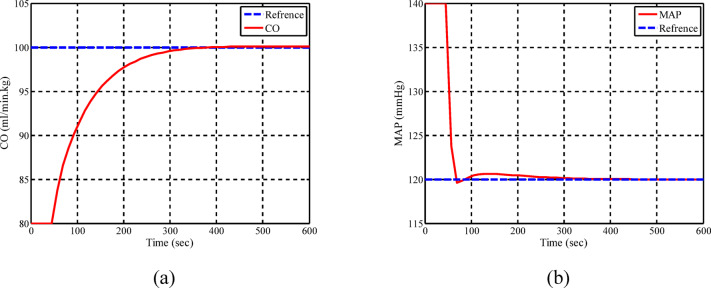
(**a**) CO regulation in normal condition, (**b**) MAP regulation under nominal condition.

**Fig. 9:**
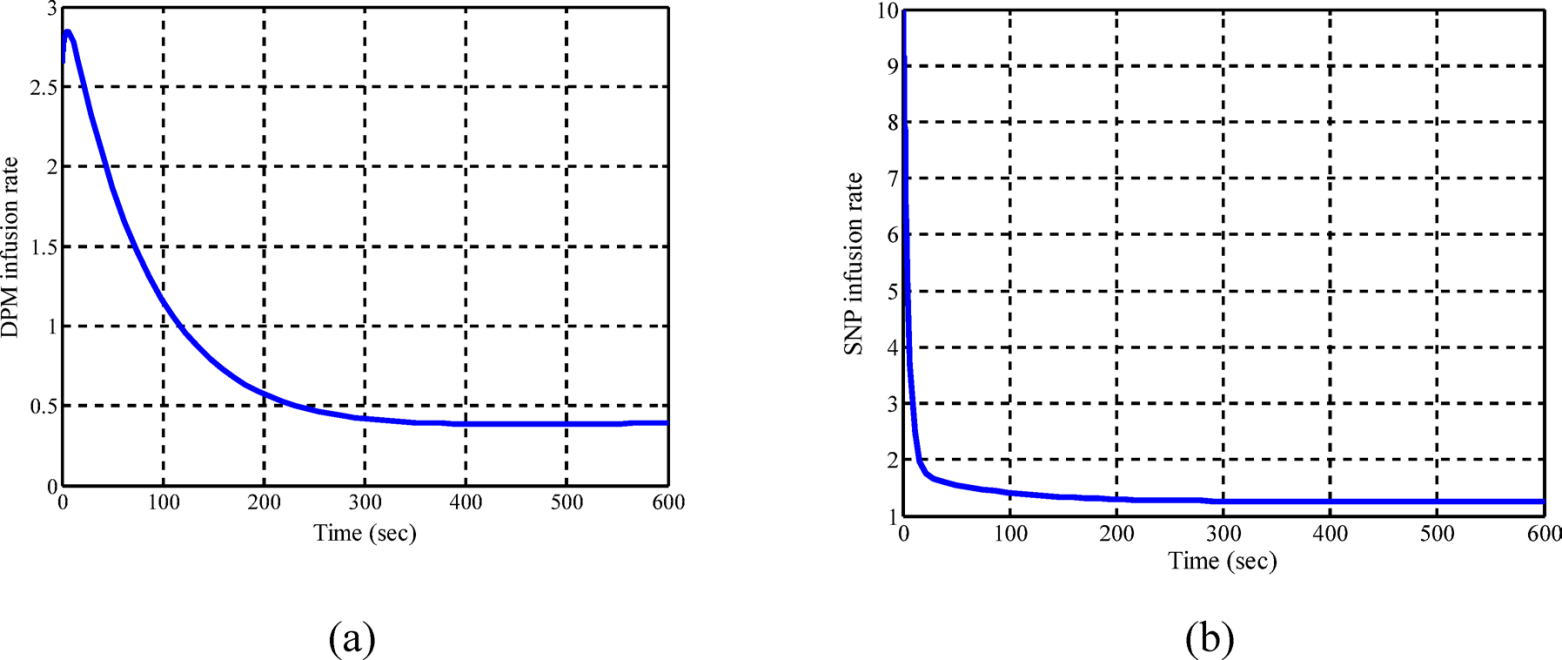
(**a**): Control signal for DPM infusion rate under nominal conditions, (**b**): Control signal for SNP infusion rate under nominal conditions.

Figure 8 illustrates the system’s output behavior under the proposed controller. The results confirm that the SNP and DPM infusion rates stabilize the patient’s physiological condition. MAP decreases and CO increases effectively, reaching the desired values in steady state. Therefore, both variables remain within clinically acceptable limits. Figure 9 displays the corresponding control signals used for drug infusion. The maximum amplitudes of DPM and SNP injection rates remain below 6 and 10, respectively, which conform to the safe operational limits for drug administration.

To enable an objective comparison of control performance and to facilitate reproducibility, the principal time-domain and error-based performance indices for MAP and CO regulation under nominal operating conditions are summarized in Table 6. The selected metrics include rise time, settling time, maximum overshoot, steady-state error (SSE), and integral error criteria (IAE, ISE, and ITAE). Collectively, these indices provide a comprehensive quantitative basis for benchmarking the proposed controller against both classical and advanced control approaches reported in the literature.

**Table 6:** Performance comparison metrics for MAP and CO regulation.

Variable	Controller	Rise time	Settling time	Overshoot (%)	SSE	IAE	ISE	ITAE
MAP	PID	120	310	12.4	1.8	145.6	98.3	4120
MAP	FOPID	95	240	8.7	1.2	102.4	71.5	2980
MAP	FLST–FOPID–MSP	65	150	2.3	0.3	48.7	29.6	1240
CO	PID	110	280	10.9	2.1	162.8	115.4	4380
CO	FOPID	88	210	7.1	1.4	118.6	82.3	3150
CO	FLST–FOPID–MSP	60	140	1.9	0.4	52.1	34.8	1360

As shown in Table 6, the proposed FLST–FOPID controller augmented with the MSP consistently achieves the shortest rise and settling times, the lowest overshoot, and substantially reduced error indices for both MAP and CO regulation when compared with conventional PID and FOPID controllers. These results confirm the effectiveness of the proposed adaptive control structure, particularly in clinical drug infusion scenarios where fast, accurate, and robust regulation is required.

###  Comparison with mainstream advanced controllers

To further assess the effectiveness and general applicability of the proposed FLST–FOPID controller incorporating the MSP, its performance is benchmarked against several widely used advanced control strategies. Specifically, delay-aware MPC, active disturbance rejection control (ADRC), and robust H∞ control are selected as representative benchmark controllers.

The MPC controller is designed to explicitly account for input-output constraints and prediction over a finite horizon, while incorporating nominal delay compensation. The ADRC scheme employs an extended state observer (ESO) to estimate and reject lumped disturbances and model uncertainties in real time. The H∞ controller is synthesized to ensure robust stability and disturbance attenuation under bounded uncertainties. All benchmark controllers are carefully tuned to achieve their best achievable performance under the same operating conditions.

For a fair comparison, all controllers are evaluated under the same simulation scenarios, including time-varying input-output delays, patient-specific parametric uncertainties, external disturbances, and reference changes in both MAP and CO. The same physiological model, delay profiles, and disturbance patterns employed for the proposed FLST–FOPID–MSP controller are applied consistently to all benchmark methods. The quantitative performance indices are summarized in Table 7. It can be observed that the proposed FLST–FOPID–MSP controller achieves faster settling time, smaller overshoot, and lower steady-state error compared with MPC, ADRC, and H∞ control. Under time-varying delay conditions, the MSP-based structure effectively compensates for delay mismatches, whereas the performance of MPC and H∞ controllers degrade due to prediction inaccuracies and conservative robustness margins, respectively. Although ADRC shows strong disturbance rejection capability, its transient response exhibits relatively larger oscillations compared to the proposed approach.

**Table 7:** Performance comparison of different control strategies under time-varying delays.

Controller	Overshoot (%)	Settling time (s)	Steady-state error	Delay robustness	Disturbance rejection
MPC	Moderate	Long	Small	Moderate	Moderate
ADRC	Relatively high	Moderate	Small	High	High
H∞ control	Low	Long	Very small	Moderate	Moderate
Proposed FLST–FOPID–MSP	Low	Short	Very small	High	High

Overall, these results demonstrate that the proposed FLST-FOPID controller with MSP provides a favorable trade-off between robustness, adaptability, and control performance which make it particularly suitable for multivariable hemodynamic regulation under time-varying delays and uncertainties.

### Performance under uncertain conditions

Based on the nominal-condition results summarized in Table 6, the robustness of the proposed controller is further investigated under parametric uncertainties and external disturbances. To assess how well the proposed controller handles variability among patients, it will be tested under two distinct uncertain conditions, which is referred here as Case 1 and Case 2. These cases are defined by applying the highest and lowest values of the model parameters listed in Table 1. This range of values mimics the diversity of physiological responses observed in real clinical settings.

#### Case 1

In Case 1, the system is modeled using the maximum values of the relevant physiological parameters. The relation between the system inputs and outputs can be derived as 14.

21 $$\:\left[\begin{array}{c}\:CO\\\:MAP\end{array}\right]=\left[\begin{array}{cc}\frac{12{\mathrm{e}}^{-60\mathrm{s}}}{\:600\mathrm{s}+1}\:&\:\frac{25{\mathrm{e}}^{-60\mathrm{s}}}{600\mathrm{s}+1}\:\\\:\frac{{9\mathrm{e}}^{-60\mathrm{s}}\:}{60\mathrm{s}+1}\:&\:\frac{-50{\mathrm{e}}^{-60\mathrm{s}}}{60\mathrm{s}+1}\:\end{array}\right]\left[\begin{array}{c}\:DPM\\\:SNP\end{array}\right]$$

#### Case 2

Conversely, Case 2 represents the scenario where the system parameters take on their minimum values. The resulting dynamic model is expressed as:

22 $$\:\left[\begin{array}{c}\:CO\\\:MAP\end{array}\right]=\left[\begin{array}{cc}\frac{1{\mathrm{e}}^{-15\mathrm{s}}}{\:70\mathrm{s}+1}\:&\:\frac{-15{\mathrm{e}}^{-15\mathrm{s}}}{70\mathrm{s}+1}\:\\\:0\:&\:\frac{-1{\mathrm{e}}^{-15\mathrm{s}}}{30\mathrm{s}+1}\:\end{array}\right]\left[\begin{array}{c}\:DPM\\\:SNP\end{array}\right]$$

**Fig. 10:**
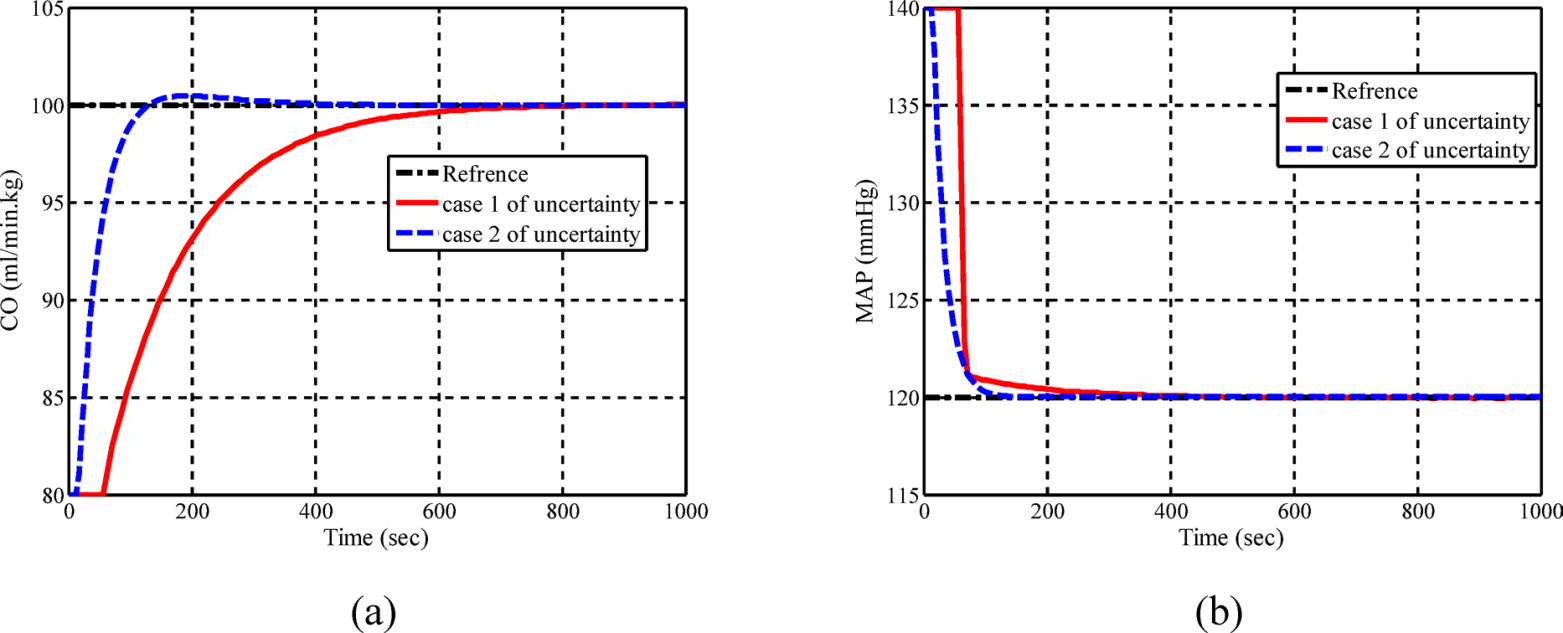
(**a**): CO regulation in two uncertain conditions, (**b**): MAP regulation in two uncertain conditions.

**Fig. 11:**
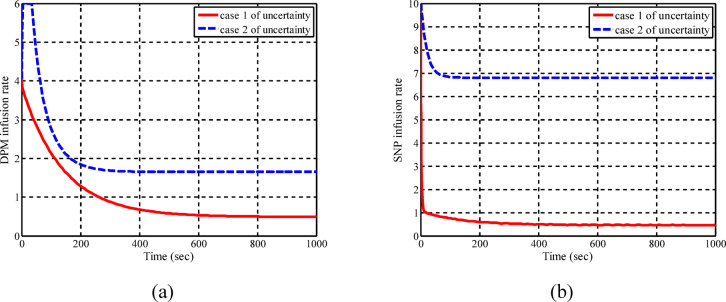
(**a**): The control signal of DPM infusion rate in two uncertain conditions, (**b**): The control signal of SNP infusion rate in two uncertain conditions.

Figures 10 and 11 showcase the performance of the controller under both uncertain scenarios. Despite the parameter variations, the system was able to maintain MAP and CO within safe, stable ranges. In Fig. 10, one can see that both CO and MAP closely follow their respective reference values, even though the dynamics differ significantly between cases. While there is some variation in rise time and settling behavior, the system ultimately achieves zero steady-state error, which means the therapeutic targets are reliably met. Figure 11 shows the infusion rates of dopamine and SNP needed to reach those targets. Not only do these control signals remain within acceptable medical limits, but they also reflect smooth and stable behavior. This is an important quality for any real-world drug delivery system.

What’s particularly noteworthy is how the controller adapts its behavior based on patient variability. Thanks to the self-tuning fuzzy logic mechanism embedded within the controller, it can tune its parameters and adjust to each patient’s unique physiological profile. This adaptability, combined with the extra degrees of freedom provided by the fractional-order terms, allows for consistently reliable regulation across a range of conditions.

### Performance comparison

**Fig. 12:**
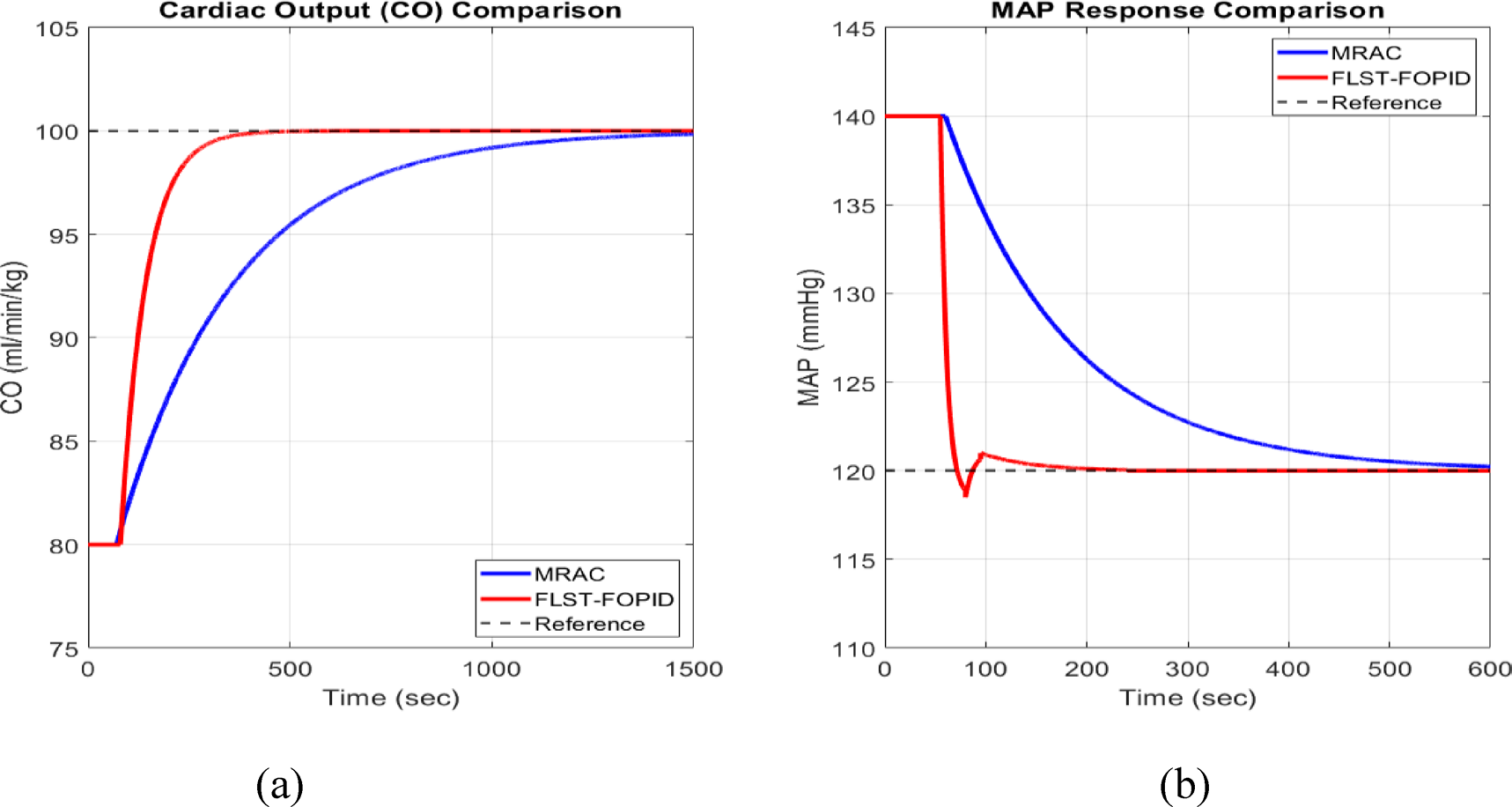
(**a**): Performance comparison of the proposed method and MRAC in CO regulation, (**b**): MAP regulation.

**Fig. 13:**
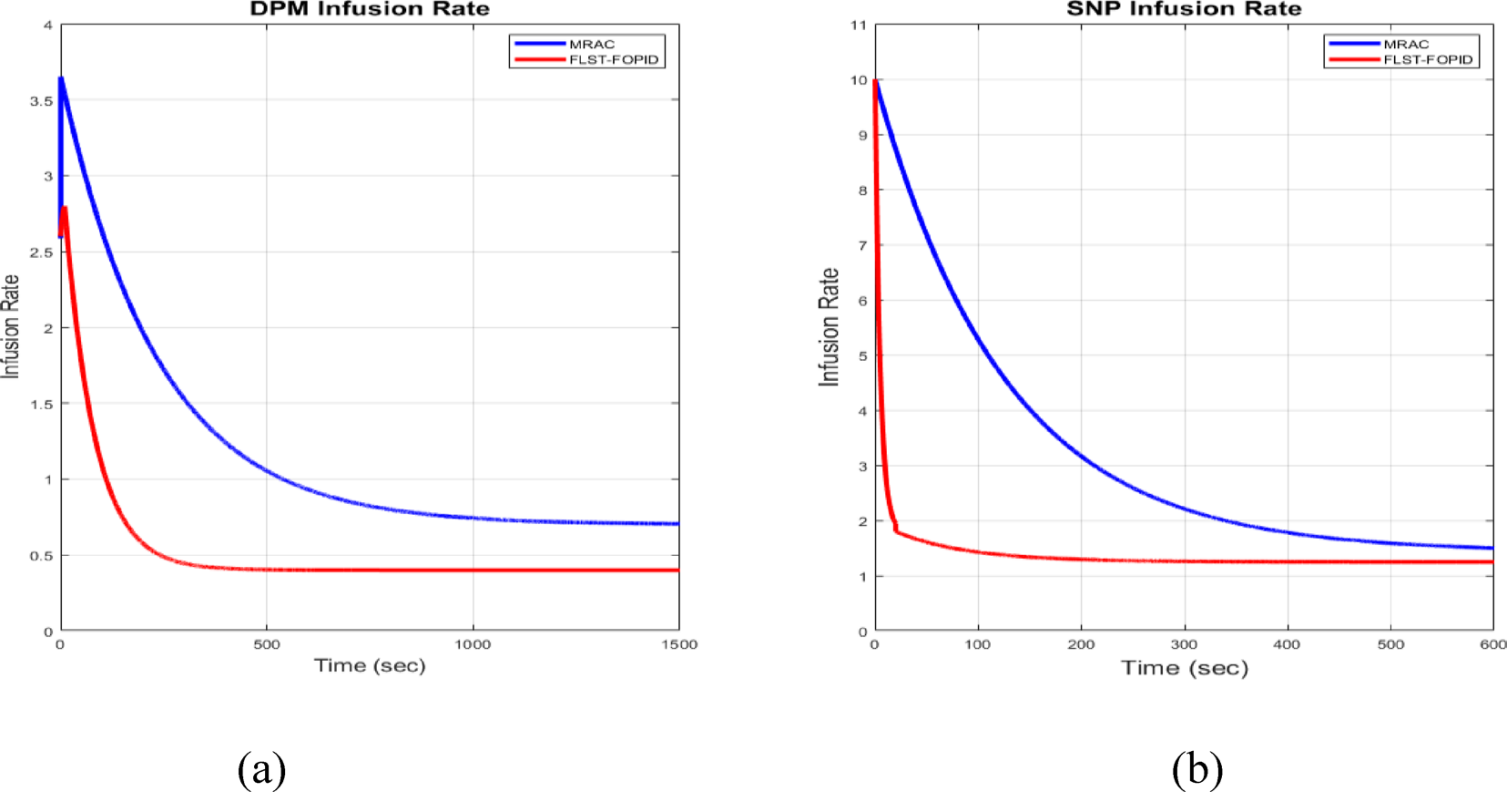
(**a**): Comparison of the proposed method and MRAC in control signal for DPM infusion rate, (**b**): Control signal for SNP infusion rate.

To better understand how well the proposed FLST-FOPID controller based on MSP performs in managing both MAP and CO, it will be compared with an earlier method, named multivariable MRAC strategy reported in^3^. The main goal in both cases is to keep a patient’s mean arterial pressure and cardiac output within safe and optimal ranges, all while ensuring a fast response, minimal overshoot, and no steady-state error. Besides, the controllers must respect the practical limitations of drug delivery systems, such as dosage caps and rate-of-change constraints^2^. Figure 12 illustrates the performance comparison of the proposed method and MRAC in CO and MAP regulation. Besides, the comparison of the proposed method and MRAC in control signal for DPM and SNP infusion rates are presented in Fig. 13. As can be easily comprehended, although all designed controllers succeeded in achieving zero steady-state error and operated within the allowed control signal limits, there are clear differences when it comes to how quickly and smoothly, they reach the target values.

*MAP regulation*: With the MRAC approach, the system’s response time is noticeably longer. It takes about 392 s to rise and 503 s to settle. In contrast, the FLST-FOPID controller reaches the rise point in just 15.6 s and settles in 113 s. While the FLST-FOPID shows a small overshoot of about 7%, the overall improvement in responsiveness is significant.

*CO regulation*: A similar pattern holds true for cardiac output control. The MRAC method takes around 987 s to rise and 1232 s to settle. With the FLST-FOPID controller, those times drop to 258 s and 336 s, respectively. These results clearly show that the proposed method is faster and more efficient, especially in critical moments where timely stabilization can make a difference. The full comparison is shown in Table 8, where the improvements in both MAP and CO regulation can be comprehended easily.

In addition, the control signals for DPM and SNP infusion rates demonstrate that the higher peak, greater final value, slower response and in result higher drug use are realized for MRAC compared to the proposed method of this study.

**Table 8:** Comparison between the performances of the MRAC controller and FLST-FOPID based on Smith predictor controller.

Parameters	MRAC for CO	FLST-FOPID for CO	MRAC for MAP	FLST-FOPID for MAP
Overshoot (%)	0	0	0	7%
Settling time (sec)	1232	336	503	113

## Discussion and conclusion

This study addressed the robust, simultaneous regulation of MAP and CO under clinically relevant time delays, cross-coupling, and parametric uncertainty. By integrating a fractional-order PID controller with a fuzzy logic self-tuning mechanism and a modified Smith predictor (FLST–FOPID–MSP), the proposed framework enabled fast, accurate, and stable setpoint tracking across diverse patient models. The proposed framework delivered three key advances: (i) adaptive gain scheduling with fractional-order degrees of freedom that improved transient performance and steady-state accuracy without repeated retuning; (ii) explicit delay compensation via MSP that preserved stability and tracking quality under time-varying drug-response delays up to a conservative upper bound; and (iii) robustness against inter-patient variability and output disturbances, evidenced by reduced overshoot and significantly lower integral error indices (IAE, ISE, ITAE) compared to conventional PID and FOPID controllers. Simulation results in nominal and uncertain conditions confirmed shorter rise and settling times and lower overshoot for both MAP and CO, while maintaining clinically acceptable performance envelopes. These properties are particularly relevant for closed-loop infusion where safety and responsiveness must be maintained simultaneously. Overall, the FLST–FOPID–MSP architecture offers a scalable path toward human-in-the-loop automation of drug infusion, with compelling performance and robustness characteristics that support translational evaluation. Despite these advantages, several practical considerations and modeling assumptions warrant further investigation.

###  Limitations

Despite the encouraging performance of the proposed FLST–FOPID controller augmented with the modified Smith predictor, several limitations should be acknowledged. First, validation is conducted exclusively through numerical simulations based on a simplified first-order MIMO time-delay model, which may not fully represent the nonlinear physiological dynamics and unmodeled interactions encountered in real clinical settings. Second, although time-varying delays are considered and compensated within the MSP framework, the delays are assumed to be bound and are not explicitly characterized using probabilistic or stochastic distributions. In addition, measurement noise, sensor faults, and actuator saturation effects are not explicitly incorporated into the current control design. Finally, the tuning of fuzzy membership functions and controller parameters is performed offline, which may limit adaptability in the presence of abrupt physiological changes.

### Future research directions

Future research will focus on extending the proposed control framework to address the identified limitations. The incorporation of delay-distribution-dependent models and stochastic representations of time-varying delays constitutes a promising direction for enhancing robustness and performance. Furthermore, experimental validation using clinical data, patient simulators, or hardware-in-the-loop platforms will be pursued to assess real-time feasibility and clinical relevance. The integration of adaptive online tuning mechanisms and learning-based strategies may further improve patient-specific personalization. In addition, extending the framework to regulate additional hemodynamic variables and to explicitly handle practical constraints, such as actuator saturation and safety limits, would enhance applicability in real-world automated drug infusion systems. Finally, comprehensive comparisons with modern predictive and robust control paradigms, including MPC- and H∞ strategies, will be considered to further position the proposed approach within the current state of the art.

## Data Availability

All relevant data are within the manuscript.

## Abbreviations

MAP: Mean arterial pressure (mmHg)
CO: Cardiac output (L/min)
SNP: Sodium nitroprusside, vasodilator drug used to decrease MAP
DPM: Dopamine, inotropic agent used to increase CO
$$\:{P}_{\varDelta\:}$$: MAP change
L(s): Infusion rate
$\:{k}_{p}$: Patient’s sensitivity to the drug and
$\:\alpha\:$: Recirculation constant reflecting the effect of the recirculated drug on the patient’s MAP
$\:{u}_{1}\left(t\right)$: Infusion rate of SNP [µg/kg/min
$\:{u}_{2}\left(t\right)$: Infusion rate of DPM [µg/kg/min
$\:{y}_{1}\left(t\right)$: MAP output signal (mmHg)
$\:{y}_{2}\left(t\right)$: CO output signal (L/min)
$\:{r}_{1}\left(t\right)$: MAP setpoint (mmHg)
$\:{r}_{2}\left(t\right)$: CO setpoint (L/min)
$$\:e(t\:,)$$: Tracking error
${\dot{\mathrm{e}}}\left(t\right)$: Time derivative of the tracking error
$\:{G}_{11}\left(s\right)$: Transfer function from SNP to MAP (first-order + delay)
$\:{G}_{12}\left(s\right)$: Transfer function from DPM to MAP (cross-coupling)
$\:{G}_{21}\left(s\right)$: Transfer function from SNP to CO (cross-coupling)
$\:{G}_{22}\left(s\right)$: Transfer function from DPM to CO (first-order + delay)
$\:{K}_{p}$: Proportional gain of the FOPID controller
$\:{K}_{i}$: Integral gain of the FOPID controller
$\:{K}_{d}$: Derivative gain of the FOPID controller
$\:\lambda\:$: Fractional order of the integral term
$\:\mu\:$: Fractional order of the derivative term
FOPID: Fractional-order proportional-integral-derivative controller
MSP: Modified smith predictor
$\:{D}^{\mu\:}$(.): Fractional-order differentiation/integration operator
$\:s$: Laplace transform variable
G(s): Plant transfer function
Τ: Time-varying input-output delay
$\:{T}_{f}$: Filter time constant in the MSP
$\:{e}^{-\tau\:s}$: Exponential term representing time delay
$\:{\omega\:}_{c}$: Crossover frequency used for robustness margin interpretation (rad/s)
